# Co-cultivation of fungal-microalgal strains in biogas slurry and biogas purification under different initial CO_2_ concentrations

**DOI:** 10.1038/s41598-018-26141-w

**Published:** 2018-05-17

**Authors:** Kai Zhou, Yuejin Zhang, Xiaobo Jia

**Affiliations:** 1 Shanghai Lianhuan Chuangmei Environmental Technology Co.Ltd., Shanghai, 200234 China; 2 College of Biological Chemical Science and Engineering, Jiaxing University, Jiaxing, 314001 China; 3 State Key Laboratory of Environmental Criteria and Risk Assessment, Chinese Research Academy of Environmental Sciences, Beijing, 100012 China

## Abstract

The effects of five different microalgae-fungi on nutrient removal and CO_2_ removal were investigated under three different CO_2_ contents (35%, 45% and 55%). The results showed that the highest nutrient and CO_2_ removal efficiency were found at 55% CO_2_ by cocultivation of different microalgae and fungi. The effect of different initial CO_2_ concentration on the removal of CO_2_ from microalgae was significant, and the order of CO_2_ removal efficiency was 55% (v/v) >45% (v/v) >35% (v/v). The best nutrient removal and biogas purification could be achieved by co-cultivation of *C*. *vulgaris* and *G*. *lucidum* with 55% initial CO_2_ content. The maximum mean COD, TN, TP and CO_2_ removal efficiency can reach 68.29%, 61.75%, 64.21% and 64.68%, respectively under this condition. All highest COD, TN, TP and CO_2_ removal efficiency were more than 85%. The analysis of energy consumption economic efficiency revealed that this strategy resulted in the highest economic efficiency. The results of this work can promote simultaneously biological purification of wastewater and biogas using microalgal-fungal symbiosis.

## Introduction

Recently, microalgae have been widely studied because of their metabolic versatility, capacity for carbon dioxide mitigation and potential applications in some areas such as wastewater treatment, and phytoremediation^1–3^. Especially, microalgae can serve as an economically and environmentally sustainable way to produce bioenergy through combining digestate decontamination and biogas purification with algal biomass production^4,5^. However, such new technical processes must be developed to capitalize on the economically feasible potential of accumulating bioproducts and biofuel inside microalgal biomass. For instance, the microalgal cell harvest from industrial cultivation for biofuel production, wastewater treatment or value-added chemicals cultivation has always been one of the major obstacles for the algae-to-fuel approach as requiring the addition of chemicals or excessive energy demand^6,7^. Up to now, all the available harvest approaches, including flocculation, flotation, centrifugal sedimentation, and filtration, have their own flaws and advantages, and usually accounts for 20–30% of the total costs of production^8^.

To resolve the major challenges facing microalgal biotechnology for harvesting, bio-flocculation has been previous performed by employing suitable microbial partner through algal-fungal interactions^9–11^. Apart from efficient harvesting of algae, co-flocculants can demonstrate their synergistic activity on total biomass, total oil production and wastewater treatment by recovering their primary nutrients, N and P, and microelements^12^. For fungal-microalgal interaction, the natural symbiosis between filamentous fungi and microalgae in the form of lichens has existed since plants evolved from green algae more than 400 million years ago and currently lichens cover 6% of the Earth’s land surface^13^. Co-cultivation of pellet forming filamentous fungi with microalgal biomass has been recently reported as efficient algal harvesting technique. Furthermore, pelletization is widely seen in the fungal fermentation process where the microorganisms are filamentous^14–16^. Even though, the detailed mechanisms of fungal-microalgal interactions remain unclear and not all filamentous fungal strains can form pellets during co-cultivations with microalgae.

CO_2_ accounts for nearly 25%–60% of the raw biogas and plays important role to simultaneously purify biogas and biogas slurry because it is used as a carbon source for the cultivation of microalgae/fungi/bacteria and converted to microalgae biomass in the presence of light by photosynthesis^17^. Meanwhile, other components of the raw biogas from anaerobic digestion is typically composed of CH_4_ (40%–75%), H_2_S (0.005%–2%), and H_2_, O_2_, or N_2_ at trace levels^2,18^ studied the biomass growth, nutrient removal, and biogas upgrading of green algae *Chlorella sp*. in a PBRb (photobioreactor bag) using LEDs (light emitting diodes) and revealed that the removal efficiency (RE) of the main nutrients were affected by the CO_2_ concentration and microalgal species, and concluded that the successful mitigation of CO_2_ and production of renewable energy (bioethanol, biogas, biodiesel, and biohydrogen) using microalgae require sorting of microalgae according to their growth rate, lipid content, and tolerance to high levels of CO_2_. Besides, most previous studies related to the cultivation of microalgae for the production of bioproducts or biodiesel and the upgrade of biogas have focused only on their growth and hydrocarbon/lipid/protein contents under low CO_2_ concentrations or ambient air^19^. Hence, it seems a significant hypothesis that appropriate selection of microalgal strains, algal-fungal and control of the influent CO_2_ concentration can achieve the optimal effect of nutrients removal and biogas upgrading.

Thus, this work focused on three objectives including: (1) appropriate selection of best fungal-microalgal interaction (i.e. *P*. *geesteranus/C*. *vulgaris*, *G*. *lucidum/C*. *vulgaris*, *P*. *ostreatus/C*. *vulgaris*, *G*. *lucidum/S*. *obliquus and G*. *lucidum/S*. *capricornutum*) for biogas slurry treatment with chemical oxygen demand (COD), nitrogen (N), phosphorus (P) and CO_2_ removal simultaneously. (2) the influence of three CO_2_ concentrations (35%, 45%, and 55%, v/v) in synthetic biogas on the nutrient removal from biogas slurry and CO_2_ removal from biogas were investigated. (3) the economic efficiency of biogas upgrading or biogas slurry nutrient removal was also evaluated according to removal efficiency, electric power charge, illumination time and LED electrical power consumption. These findings are essential for the effective design of crude biogas treatment systems, in order to address the demands of technology efficiency and environmental sustainability.

## Results and Discussion

### The five selected strains growth at different CO_2_ concentration

These five fungal-microalgal mixture strains survived all treatments. Table 1 shows the results of cell growth and average daily productivity of the selected five different fungal-microalgal mixture (i.e., *P*. *geesteranus/C*. *vulgaris*, *G*. *lucidum/C*. *vulgaris*, *P*. *ostreatus/C*. *vulgaris*, *G*. *lucidum/S*. *obliquus and G*. *lucidum/S*. *capricornutum*) under different CO_2_ concentration treatments (35%, 45% and 55%, v/v). From Table 1, it was concluded that the growth rates under 55% CO_2_ concentration treatment were higher than 45% and 35% CO_2_ concentration treatment. Probably reason is that biomass production of the fungal-microalgal mixture depends on CO_2_ consumption as the carbon source under phototropic condition^20^. This statement can also be supported by the results of the mean daily productivity data shown in Table 1. Notably, the growth rate and mean daily productivity under 55% CO_2_ concentration were recorded highest by *G*. *lucidum/C*. *vulgaris* mixture (0.352 d^−1^, 0.174 gL^−1^d^−1^), follow by *G*. *lucidum/S*. *obliquus*, *P*. *geesteranus/C*. *vulgaris*, *P*. *ostreatus/C*. *vulgaris and G*. *lucidum/S*. *capricornutum*. Hence, high CO_2_ concentration (55%, v/v) was chosen as the most effective treatment and *G*. *lucidum/C*. *vulgaris* strain can be ranked as the optimal fungal-microalgal mixture according to its high biomass production.

**Table 1:** Growth rates and mean daily productivity of the five selected strains under different CO_2_ concentration treatments.

The selected strains/CO_2_ concentration	CO_2_ 35% (v/v)	CO_2_ 45% (v/v)	CO_2_ 55% (v/v)
	Growth rate d^−1^		
*P*. *geesteranus/C*. *vulgaris*	0.268 ± 0.05	0.292 ± 0.06	0.308 ± 0.06
*G*. *lucidum/C*. *vulgaris*	0.309 ± 0.06	0.336 ± 0.07	0.352 ± 0.06
*P*. *ostreatus/C*. *vulgaris*	0.241 ± 0.05	0.271 ± 0.07	0.287 ± 0.05
*G*. *lucidum/S*. *obliquus*	0.296 ± 0.05	0.314 ± 0.06	0.321 ± 0.07
*G*. *lucidum/S*. *capricornutum*	0.229 ± 0.04	0.255 ± 0.05	0.273 ± 0.05
	Mean daily productivity(gL^−1^ d^−1^)		
*P*. *geesteranus/C*. *vulgaris*	0.106 ± 0.006	0.115 ± 0.012	0.131 ± 0.006
*G*. *lucidum/C*. *vulgaris*	0.132 ± 0.007	0.153 ± 0.014	0.174 ± 0.008
*P*. *ostreatus/C*. *vulgaris*	0.094 ± 0.005	0.105 ± 0.009	0.112 ± 0.006
*G*. *lucidum/S*. *obliquus*	0.118 ± 0.007	0.132 ± 0.011	0.153 ± 0.007
*G*. *lucidum/S*. *capricornutum*	0.081 ± 0.006	0.098 ± 0.008	0.107 ± 0.006

However, what were the possible reasons that *G*. *lucidum/C*. *vulgaris* co-cultivation mixture was superior to the other four similar mixtures? Firstly, biomass production ability of microalgae strain *C*. *vulgaris* was relatively high than some other microalgae based on previous studies^21–25^. For example, Zhao *et al*.^21,23^ reported that the growth rate and mean daily productivity of high-yield strain *C*. *vulgaris* can reach 0.363 d^−1^ and 0.112 g L^−1^ d^−1^ with optimal wavelength mixing ratios treatments, and reach 0.372 d^−1^ and 0.183 g L^−1^ d^−1^ with treatment of synthetic high-strength wastewater^21,23^. Secondly, based on the molecular mechanism of filamentous fungal-based bio-flocculation, fungal cell capacity for self-pelletization may be significantly different as it is strain-specific and not all filamentous fungal strains can form pellets during growth^26^. In this study, after co-cultivation, green-colored pellets were found by interaction between fungal strain *G*. *lucidum* and microalgal strain *C*. *vulgaris*, instead of milky white-colored pellets like other four fungal-microalgal mixture, which indicate that pelletization capacity of *G*. *lucidum/C*. *vulgaris* mixture was relatively strong. It is unavoidable to form biofilm on the wall of reactor, the final treatment efficiencies will be affected by some parameters such as the decreased illumination intensity or shortage of nutrient^27^. However, according to our experiences^28,29^, algal-fungal symbionts achieved relatively high biomass for 10 days and the removal efficiencies of pollutants decreased after 10 days, which will lead to poor economic efficiency if last for a longer period of cultivation time.

For *P*. *geesteranus/C*. *vulgaris*, and *P*. *ostreatus/C*. *vulgaris* mixtures, simple adherence or entrapment mode was found for the interaction between microalgal cells and the fungal pellets^30^. Likewise, Linder^31^ reported that fungal cell capacity for self-pelletization was correlated to the accumulation of a family of low molecular weight amphipathic, hydrophobic proteins accumulated on the hyphal surface^31^. These hydrophobic proteins are potentially involved in hyphae adherence to solid substrates^32^. Thirdly, pH was the key factor affecting formation of fungal-algal pellet^15,20^. For *G*. *lucidum*/*C*.*vulgaris* mixture under 55% CO_2_ concentration treatment in the experiment, final pH value was 7.16 after 10 days co-cultivation period, which is slightly high than the other four fungal-microalgal mixtures. Therefore, enhanced solubility of CO_2_ in the alkalescent biogas slurry was found, which act as the carbon source in the nutrient solution. It is similar to the previous conclusion that pH serviced as the key factor to induce the pelletization of *M*. *circillenous* alone^33^. Likewise, Liu *et al*.^34^ used pH adjustment to induce the formation of fungal cell pelletization, providing a simplified method by which to facilitate the cell harvest of oleaginous cells^34^.

### Nutrient removal efficiencies at different CO_2_ concentration

**Figure 1:**
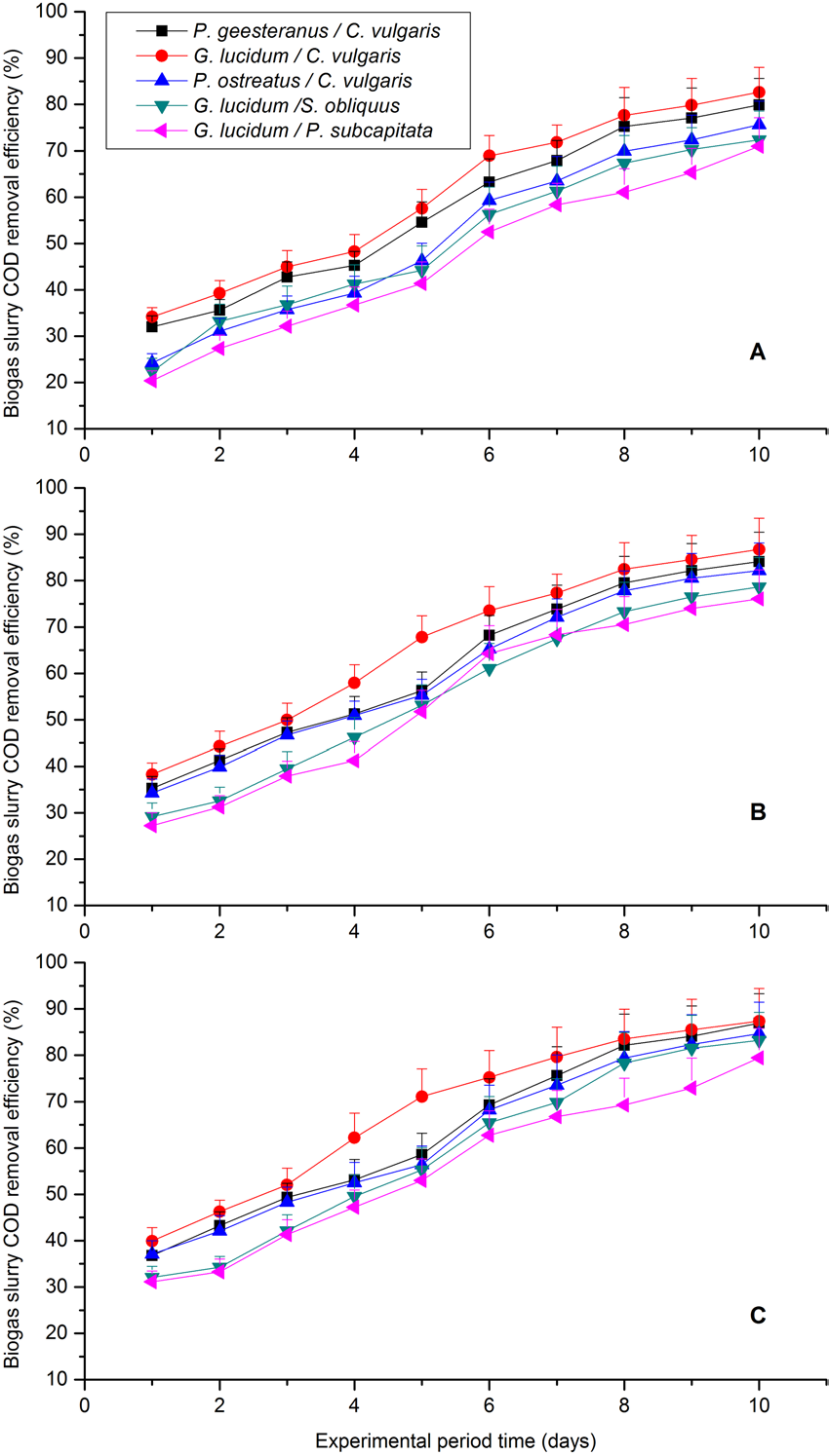
COD removal efficiency with time under different CO_2_ concentrations for the five selected microalgal-fungal strains: (**A**) 35% (v/v) CO_2_, (**B**) 45% (v/v) CO_2_, and (**C**) 55% (v/v) CO_2_.

Based on Table 1, a considerably high average COD remove efficiency (55.72%–68.29%) was achieved with 55% CO_2_ concentration treatment, followed by 54.26%–66.29% and 46.62%–60.52% with 45% and 35% CO_2_ concentration treatments. This variation trend can further confirmed the conclusion that organic carbon is the basic ingredient of microalgae, which accounts for about half of microalgal biomass and can be utilize for heterotrophic or mixotrophic growth^35–37^. In this study, under 55% CO_2_ concentration treatment, the five fungal-microalgal strains grown at autotrophic and heterotrophic conditions using CO_2_ as the only carbon source. Besides, the corresponding average COD remove efficiency ranked: *G*. *lucidum/C*. *vulgaris* (68.29%) > *P*. *geesteranus/C*. *vulgaris* (63.92%) > *P*. *ostreatus/C*. *vulgaris* (62.45%) > *G*. *lucidum/S*. *obliquus* (59.17%) > *G*. *lucidum/S*. *capricornutum* (55.72%). Furthermore, Fig. 1 depicts the changes in COD removal during 10 days of the experimental period and maximum COD removal efficiency even reached 87.37% by *G*. *lucidum/C*. *vulgaris*, which is slightly high than most previous report conclusions. For instance, Zhao *et al*.^38^ demonstrated that the highest COD removal efficiency can reach 85.35%^38^. Similarly, Yan and Zheng^18,39^ carried out series researches and reported that 86% of COD could be removed by *Chlorella sp*. within 24 h with optimal photoperiods, while 78.9% with optimal mixed wavelength ratio (red:blue = 5:5)^18,39^. These different results are closely related to different influent CO_2_ concentrations, photoperiod of the experiment. Besides, all the results imply that the screening of microalgal, fungal and fungal-microalgal strains is effective to reduce the COD in biogas slurry.

**Figure 2:**
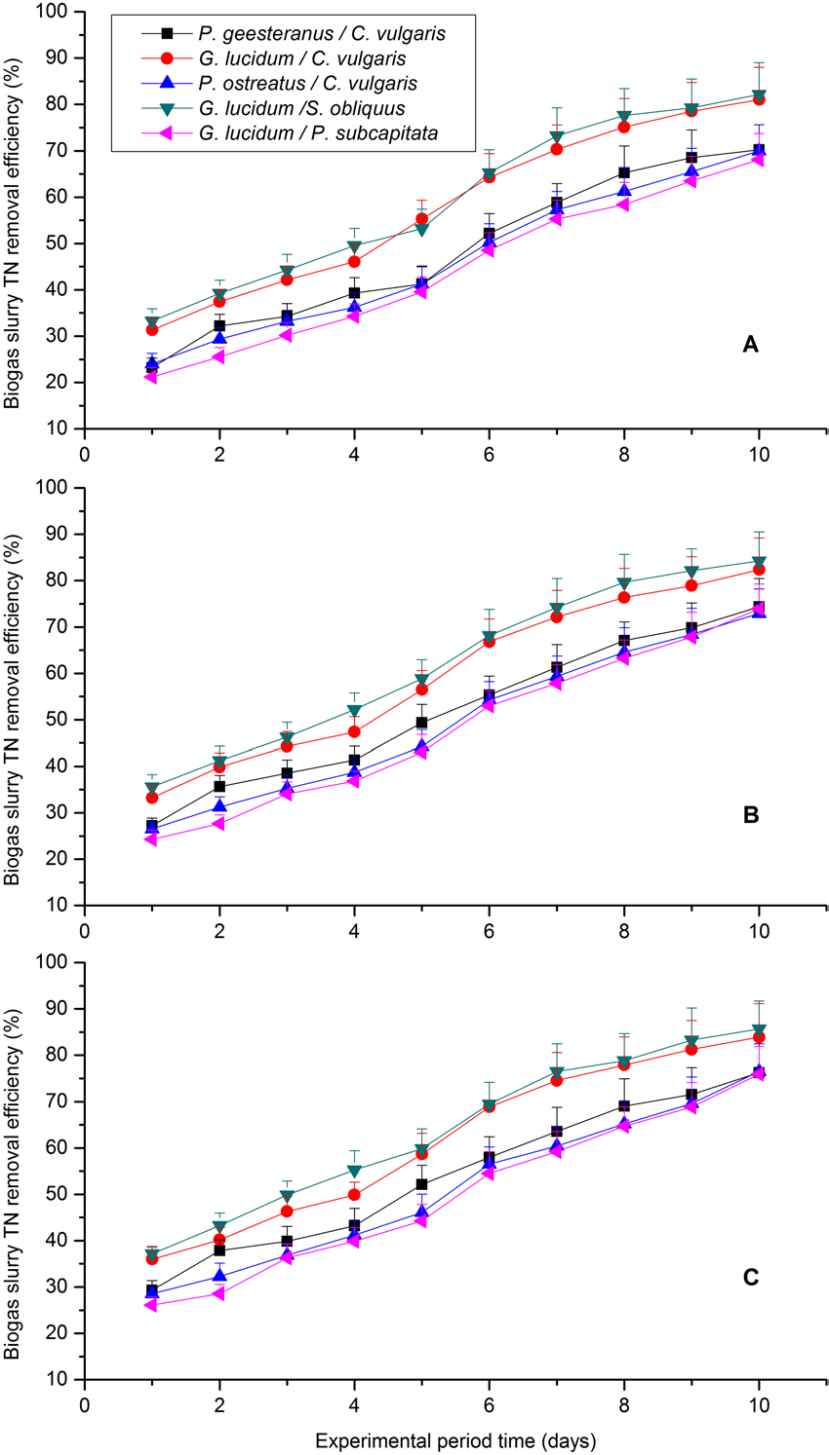
TN removal efficiency with time under different CO_2_ concentrations for the five selected microalgal-fungal strains: (**A**) 35% (v/v) CO_2_, (**B**) 45% (v/v) CO_2_, and (**C**) 55% (v/v) CO_2_.

According to Table 2, TN in the biogas slurry was removed by the five fungal-microalgal strains significantly under the three CO_2_ concentration treatments. But the TN removal efficiencies were a little different during 10 days experimental schedule as displayed in Fig. 2. The highest average TN remove efficiency was obtained under 55% CO_2_ concentration treatment for the five fungal-microalgal mixtures containing *P*. *geesteranus/C*. *vulgaris*, *G*. *lucidum/C*. *vulgaris*, *P*.*ostreatus/C*. *vulgaris*, *G*. *lucidum/S*. *obliquus and G*. *lucidum/S*. *capricornutum*, reaching 54.07%, 61.75%, 51.32%, 63.93% and 59.83%, respectively. Especially, the mixed culture containing *G*. *lucidum/S*. *obliquus* demonstrated highest N removal efficiency, while *P*.*ostreatus/C*. *vulgaris* showed relatively low TN removal efficiency. Coincidentally, it showed very consistent with previous reported conclusion that the highest TN remove efficiency was obtained for the bacterial-microalgal mixture containing *C*. *vulgaris*, *S*. *obliquus*, and *N*.*oleoabundans*, reaching 61.49%, 63.13%, and 55.26%, respectively^2^. In addition, Zhao *et al*.^21^ also recommended that *S*.*obliquus* can contribute to a high nitrogen RE than *C*. *vulgaris* or *N*. *oleoabundans* and nitrogen RE even reached 76% given appropriate mixed ratio of wavelength (red:blue = 7:3)^21^. Xu *et al*.^40^ investigated an integrated approach that combined freshwater microalgae *Scenedesmus obliquus* cultivation with piggery anaerobic digestate liquid treatment and revealed that average nitrogen RE was 58.39–74.63%^40^. Assimilation within microalgal biomass may be the key mechanism of nitrogen removal because reproduction of fungal/microalgal need sufficient nitrogen source to produce nucleic acids^41^.

**Table 2:** Average removal and the economic efficiency of biogas CO_2_ and biogas slurry nutrient reduction under the three CO_2_ concentrations.

Five strains*/*CO_2_ concentration treatments	Removal efficiency (%)				Economic efficiency (USD^−1^)			
	COD	TN	TP	CO_2_	COD	TN	TP	CO_2_
CO_2_ 35% (v/v)								
*P*. *geesteranus/C*. *vulgaris*	57.37 ± 4.02^a^	48.53 ± 4.24^b^	51.29 ± 3.62^b^	54.15 ± 4.12^a^	29.56 ± 1.92^ab^	20.25 ± 1.75^b^	27.35 ± 2.16^b^	26. 35 ± 2.34^a^
*G*. *lucidum/C*. *vulgaris*	60.52 ± 4.71^a^	58.15 ± 4.79^a^	59.37 ± 4.16^a^	57.18 ± 4.33^a^	31.24 ± 2.21^a^	29.98 ± 2.04^a^	30.34 ± 2.35^a^	28.19 ± 2.57^a^
*P*. *ostreatus/C*. *vulgaris*	51.72 ± 3.65^b^	46.84 ± 3.22^bc^	49.35 ± 3.25^b^	49.22 ± 3.68^b^	27.96 ± 2.06^b^	19.41 ± 1.62^b^	21.32 ± 1.73^c^	22.68 ± 1.91^b^
*G*. *lucidum/S*. *obliquus*	50.53 ± 3.24^b^	59.71 ± 4.83^a^	57.64 ± 4.37^a^	47.43 ± 3.95^b^	26.35 ± 1.88^b^	30.67 ± 2.13^a^	28.51 ± 2.24^ab^	20.54 ± 1.79^b^
*G*. *lucidum/S*. *capricornutum*	46.62 ± 3.59^c^	44.45 ± 3.06^c^	58.08 ± 4.69^a^	43.36 ± 3.56^c^	19.25 ± 1.27^c^	18.02 ± 1.45^b^	28.98 ± 2.62^ab^	17.44 ± 1.35^c^
CO_2_ 45% (v/v)								
*P*. *geesteranus/C*. *vulgaris*	61.92 ± 4.13^b^	52.01 ± 4.39^c^	54.07 ± 4.37^b^	59.26 ± 4.64^b^	32.45 ± 2.73^b^	28.12 ± 2.63^ab^	29.26 ± 2.19^b^	30. 91 ± 2.68^a^
*G*. *lucidum/C*. *vulgaris*	66.29 ± 4.27^a^	59.78 ± 4.68^b^	61.75 ± 4.23^a^	63.07 ± 5.12^a^	35.87 ± 2.98^a^	30.86 ± 2.79^ab^	32.81 ± 2.31^a^	32.76 ± 2.84^a^
*P*. *ostreatus/C*. *vulgaris*	60.51 ± 3.84^b^	49.56 ± 4.13^cd^	51.32 ± 4.15^bc^	58.04 ± 4.39^b^	31.13 ± 2.34^b^	21.37 ± 1.75^d^	27.26 ± 2.12^bc^	29.91 ± 2.12^ab^
*G*. *lucidum/S*. *obliquus*	55.76 ± 3.92^c^	62.26 ± 5.02^a^	63.91 ± 5.07^a^	52.47 ± 4.61^c^	28.37 ± 2.43^bc^	32.61 ± 2.33^a^	33.19 ± 2.37^a^	28.39 ± 2.04^b^
*G*. *lucidum/S*. *capricornutum*	54.26 ± 3.78^c^	48.18 ± 3.96^d^	49.83 ± 3.91^c^	51.61 ± 4.48^c^	27.18 ± 2.06^c^	24.83 ± 2.02^c^	25.35 ± 2.18^c^	27.25 ± 2.27^b^
CO_2_ 55% (v/v)								
*P*. *geesteranus/C*. *vulgaris*	63.92 ± 5.02^b^	54.07 ± 4.37^b^	56.29 ± 4.32^b^	60.83 ± 5.34^b^	33.04 ± 2.35^b^	26.79 ± 2.15^b^	28.14 ± 2.01^b^	31.84 ± 2.63^ab^
*G*. *lucidum/C*. *vulgaris*	68.29 ± 4.73^a^	61.75 ± 4.68^a^	64.21 ± 5.36^a^	64.68 ± 5.61^a^	37.17 ± 2.93^a^	32.21 ± 2.54^a^	34.05 ± 2.62^a^	34.19 ± 2.71^a^
*P*. *ostreatus/C*. *vulgaris*	62.45 ± 4.57^bc^	51.32 ± 4.05^b^	53.74 ± 4.41^bc^	58.53 ± 4.87^bc^	32.86 ± 2.56^b^	26.83 ± 2.19^b^	28.71 ± 2.13^b^	29.34 ± 2.26^bc^
*G*. *lucidum/S*. *obliquus*	59.17 ± 4.16^c^	63.93 ± 5.13^a^	61.98 ± 5.34^a^	55.62 ± 4.63^cd^	30.73 ± 2.88^b^	33.34 ± 2.78^a^	32.87 ± 2.66^a^	27.63 ± 2.11^c^
*G*. *lucidum/S*. *capricornutum*	55.72 ± 4.02^bc^	59.83 ± 5.04^a^	52.29 ± 4.79^c^	54.84 ± 3.99^d^	27.85 ± 2.29^c^	31.18 ± 2.71^a^	28.67 ± 2.35^b^	27.59 ± 2.38^c^

Note: Values with different superscript letters demonstrate a significant difference at level of *p* < 0.05 for the same CO_2_ concentration according to the Duncan’s multiple range tests.

**Figure 3:**
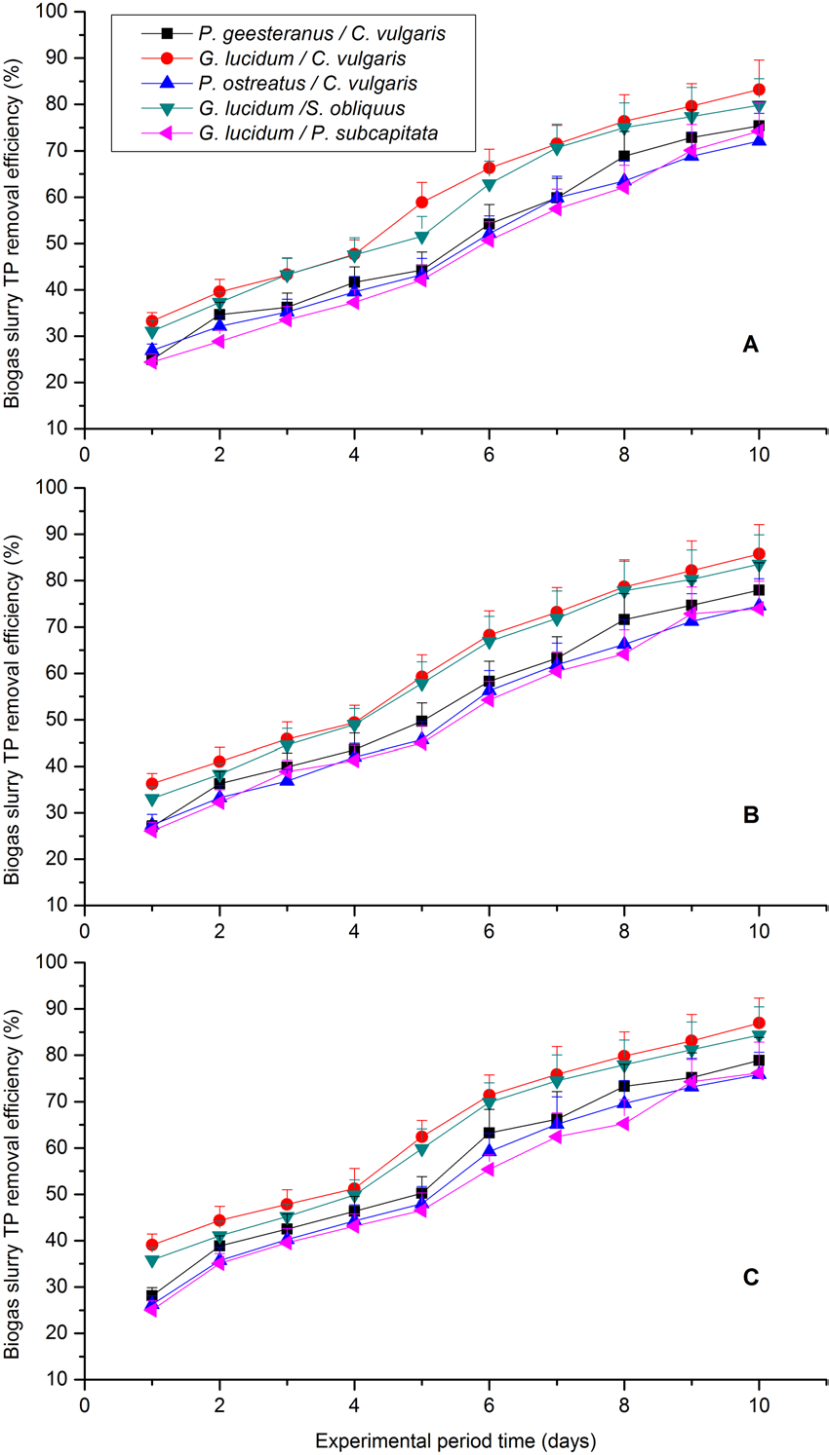
TP removal efficiency with time under different CO_2_ concentrations for the five selected microalgal-fungal strains: (**A**) 35% (v/v) CO_2_, (**B**) 45% (v/v) CO_2_, and (**C**) 55% (v/v) CO_2_.

As far as phosphorus was concerned, five fungal-microalgal mixtures survived in the co-cultivation and phosphorus REs were recorded as 56.29%, 64.21%, 53.74%, 61.98% and 52.29%, respectively, with 55% CO_2_ concentration treatment, which is higher than under 45% and 35% CO_2_ concentration treatment. Obviously, the trend of TP removal efficiency (Fig. 3) is consistent with those of fungal-microalgal growth rate, but not the same to COD and nitrogen RE (Table 1 and Table 2). Phosphorus is a key element in microalgae culture and is an important component of cell membrane phospholipids and adenosine triphosphate^22^. Furthermore, the presence of Ca^2+^ and Mg^2+^ in the biogas slurry and the alkaline conditions caused by fungal-microalgal growth promoted phosphorus precipitation and the formed deposits were helpful for phosphorus removal from biogas slurry^42,43^. Zhao *et al*.^23^ reported that the TP RE by the three microalgae (i.e., *C*. *vulgaris*, S. *capricornutum*, and *S*. *obliquus*) was 97.01%, 95.40%, and 95.87% for high C loading waste water with initial P concentration of 0.4 mg L^−1 ^^23^. Similarly, Powell^44^ reported that more than 95% of the soluble P in the primary effluent was removed by *Chlorella* when the initial P concentrations were 4 mg L^−1^ for the primary effluent^44^. It is worth noting that the phosphorus RE observed in this work with fungal-algal mixture seems slightly lower than above-mentioned previous works. Most significant influence factor for such strange phenomenon was the initial phosphate concentration in the biogas slurry, which had a strong influence on the accumulation of polyphosphate in the microalgae. Thus, the high initial phosphate concentration (20 mg L^−1^) was probably responsible for the uncompleted consumption of TP in this study^23,44^.

Above all, the biogas slurry nutrient (i.e., COD, TN, and TP) were reduced efficiently and similarly by the five fungal-microalgal mixtures under the different CO_2_ concentration treatments (35%, 45% and 55%, v/v) for 10 days batch culture, and the nutrient remove efficiency (REs) were presented in Table 2 and Figs 1–3 The nutrient removal efficiencies with 55% CO_2_ treatments were higher than those of the other treatments and achieved the highest COD, TN, and TP removal efficiencies of 68.29 ± 4.73%, 63.93 ± 5.13%, and 64.21 ± 5.36%, respectively. However, no consistent relationship was found between COD remove efficiency, TN remove efficiency, TP remove efficiency and fungal/microalgal growth rates in this study. In other words, the fungal-microalgal growth rate is not proportional to the nutrients (COD, TN and TP) removal from biogas slurry, which is consistent with the conclusion previous reported before by Yan *et al*.^39^ and Wang *et al*.^22^. These nutrient removals were mainly achieved via an assimilation process of microalgal/fungal reproduction as they require abundant carbon, nitrogen, and phosphorous sources for heterotrophic or mixotrophic growth^22,36^. But, it was not consistent for COD, TN and TP remove efficiency as detailed mechanism was concerned. Therefore, selection and optimization of fungal-microalgal strains is very important for biogas upgrading, biogas slurry nutrients removal, microalgal metabolism and greenhouse gas reduction. In this study, the optimal fungal-microalgal mixture for biogas slurry nutrients removal was *G*. *lucidum/C*. *vulgaris* under 55% CO_2_ concentration treatments.

### Biogas upgrading

**Figure 4:**
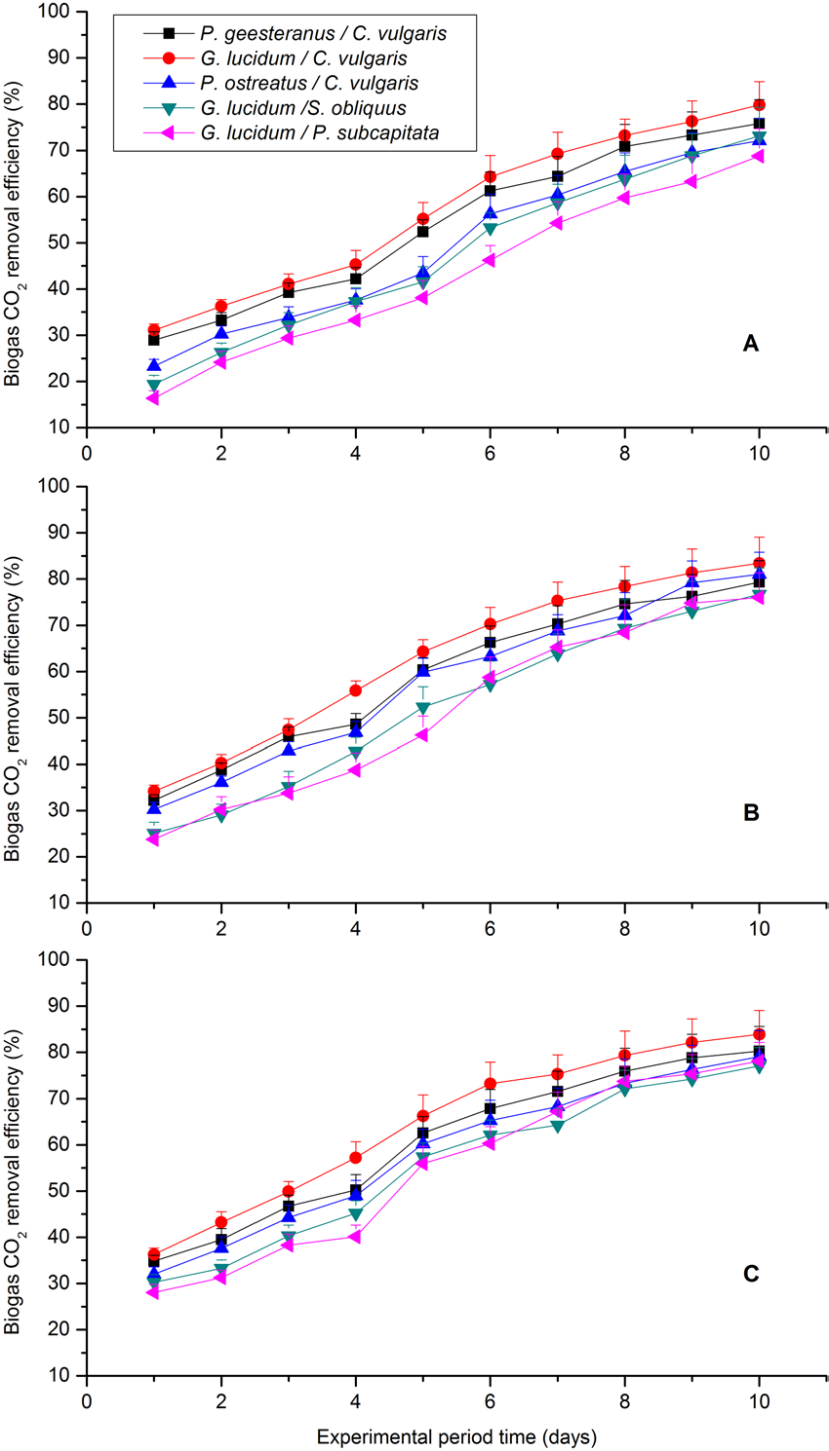
CO_2_ removal efficiency with time under different CO_2_ concentrations for the five selected microalgal-fungal strains: (**A**) 35% (v/v) CO_2_, (**B**) 45% (v/v) CO_2_, and (**C**) 55% (v/v) CO_2_.

Average CO_2_ removal rates (%) were investigated as a function of operating time to evaluate differences in biogas upgrading with different CO_2_ influent concentrations for the five fungal-microalgal strains (Table 2, Fig. 4). Specifically, *G*. *lucidum/C*. *vulgaris* strain recorded high average CO_2_ removal rate of 64.21 ± 5.36%, followed by *P*. *geesteranus/C*. *vulgaris* for 64.21 ± 5.34%, *P*. *ostreatus/C*.*vulgaris* for 58.53 ± 4.87%, *G*. *lucidum/S*. *obliquus* for 55.62 ± 4.63% and *G*. *lucidum/S*. *capricornutum* for 54.84 ± 3.99%. This result agrees with the findings of previous studies by Sun *et al*.^2^, who reported that CO_2_ can be reduced up to 49.95%–62.31% by bacterial-microalgal co-cultivation containing *S*. *obliquus*, *C*. *vulgaris*, *N*. *oleoabundans* and activated sludge^2^. At the end of experimental duration, the highest CO_2_-remove efficiency was recorded as 86.97 ± 5.38% by *G*. *lucidum/C*. *vulgaris* strain (Fig. 4), which was higher than most conclusions that reported before^18,21,23,39^. It can further confirmed that selection and optimization of fungal-microalgal strain can significantly address such issues as CO_2_ sequestration, biomass production, nutrient removal of biogas slurry, and simultaneously biogas purification for engineering progress in the future. Moreover, the effect of biogas upgrading in this study agreed with the variation trends of the growth rates and mean daily productivity for the fungal-microalgal strains (Table 1). Half of such biomass reproduction was derived from CO_2_ sequestration^45^. If took algal-fungal biomass production based on 1 Kg CO_2_ removal as a measurable indicator, *G*. *lucidum/C*. *vulgaris* had the highest the biomass production, which were 644.33 g/L, 529.76 g/L and 484.24 g/L, respectively. *G*. *lucidum/S*. *capricornutum* had the lowest biomass production, which was consistent with the analysis of the growth characteristics of the algae. In addition, all the algal-fungal biomass production decreased as initial CO_2_ concentration increased from 35% to 55%. This finding implied that high CO_2_ could inhibit growth of the algal-fungal biomass. This is consist with Sun’s research^2^.

Though numerous influence factors, such as mix wavelength ratio, photosynthetic photon flux density, different photoperiod treatments, initial CO_2_ influent concentrations, algal strains and different C/N ratios, were expatiated deeply for reduction of CO_2_ in the biogas, investigation about effect of pH for biogas upgrading was still incomplete in the previous research^2,18,21–23,38,39^. In this study, pH was detected and recorded every day during 10 days experimental duration for every fungal-microalgal strain under all initial CO_2_ influent concentration (Table 3). Similar variation trend of pH was found and was proportion to biomass reproduction and biogas slurry nutrients removal. It was induced that elevated pH or slight alkalescent biogas slurry contributed to enhance sequestration of CO_2_ by solution of assimilation. In addition, O_2_ and H_2_O (v/v) concentrations in the biogas almost unvaried during the experimental period (data not shown). O_2_ concentration (v/v) was increased from 0.12% ± 0.02% to 0.52% ± 0.04% (data not shown), whereas H_2_O concentrations (v/v) were in the range of 1.12% ± 0.16% and 3.03% ± 0.22% (data not shown). Since raw biogas always contains saturated steam, the presence of H_2_O in the upgraded biogas does not negatively affect the growth of microalgae. In addition, H_2_O and O_2_ can also be applied for microalgal photosynthesis and respiration^46^.

**Table 3:** Variations in pH under various CO_2_ concentration treatments for the five selected strains.

Co-cultivation types/CO_2_ concentration treatments	Time(h)										
	0	24	48	72	96	120	144	168	192	216	240
CO_2_ 35% (v/v)											
*P*. *geesteranus/C*. *vulgaris*	6.84 ± 0.07	6.86 ± 0.11	6.89 ± 0.15	6.92 ± 0.18	6.95 ± 0.19	6.98 ± 0.21	7.01 ± 0.23	7.04 ± 0.18	7.06 ± 0.26	7.11 ± 0.21	7.13 ± 0.27
*G*. *lucidum/C*. *vulgaris*	6.86 ± 0.13	6.89 ± 0.15	6.92 ± 0.18	6.95 ± 0.16	6.98 ± 0.21	7.02 ± 0.23	7.05 ± 0.25	7.08 ± 0.22	7.11 ± 0.24	7.13 ± 0.21	7.14 ± 0.23
*P*. *ostreatus/C*. *vulgaris*	6.85 ± 0.11	6.88 ± 0.13	6.94 ± 0.16	6.98 ± 0.17	7.01 ± 0.19	7.04 ± 0.21	7.07 ± 0.24	7.09 ± 0.22	7.13 ± 0.26	7.12 ± 0.19	7.15 ± 0.29
*G*. *lucidum/S*. *obliquus*	6.87 ± 0.12	6.92 ± 0.15	6.94 ± 0.22	6.96 ± 0.24	6.99 ± 0.23	7.03 ± 0.24	7.06 ± 0.26	7.11 ± 0.27	7.12 ± 0.28	7.15 ± 0.25	7.17 ± 0.32
*G*. *lucidum/S*. *capricornutum*	6.89 ± 0.15	6.93 ± 0.21	6.97 ± 0.23	7.01 ± 0.26	7.04 ± 0.22	7.06 ± 0.19	7.09 ± 0.27	7.12 ± 0.21	7.15 ± 0.24	7.18 ± 0.29	7.13 ± 0.25
CO_2_ 45% (v/v)											
*P*. *geesteranus/C*. *vulgaris*	6.88 ± 0.11	6.89 ± 0.14	6.92 ± 0.17	6.94 ± 0.19	6.97 ± 0.21	6.98 ± 0.25	7.02 ± 0.24	7.04 ± 0.27	7.07 ± 0.23	7.12 ± 0.24	7.14 ± 0.28
*G*. *lucidum/C*. *vulgaris*	6.83 ± 0.15	6.87 ± 0.17	6.91 ± 0.19	6.93 ± 0.22	6.95 ± 0.21	7.01 ± 0.24	7.04 ± 0.25	7.06 ± 0.22	7.08 ± 0.25	7.11 ± 0.27	7.13 ± 0.31
*P*. *ostreatus/C*. *vulgaris*	6.84 ± 0.12	6.86 ± 0.21	6.89 ± 0.24	6.93 ± 0.25	6.98 ± 0.25	7.02 ± 0.26	7.05 ± 0.24	7.07 ± 0.26	7.09 ± 0.28	7.13 ± 0.23	7.15 ± 0.25
*G*. *lucidum/S*. *obliquus*	6.89 ± 0.13	6.93 ± 0.18	6.96 ± 0.21	7.01 ± 0.24	7.04 ± 0.23	7.01 ± 0.25	7.07 ± 0.27	7.11 ± 0.24	7.13 ± 0.29	7.15 ± 0.26	7.17 ± 0.31
*G*. *lucidum/S*. *capricornutum*	6.82 ± 0.16	6.87 ± 0.19	6.92 ± 0.22	6.95 ± 0.23	7.01 ± 0.22	7.05 ± 0.27	7.09 ± 0.23	7.12 ± 0.29	7.15 ± 0.26	7.11 ± 0.23	7.16 ± 0.29
CO_2_ 55% (v/v)											
*P*. *geesteranus/C*. *vulgaris*	6.79 ± 0.16	6.86 ± 0.17	6.89 ± 0.19	6.95 ± 0.21	6.97 ± 0.24	6.99 ± 0.23	7.02 ± 0.21	7.05 ± 0.27	7.08 ± 0.26	7.12 ± 0.28	7.15 ± 0.27
*G*. *lucidum/C*. *vulgaris*	6.89 ± 0.19	6.92 ± 0.22	6.94 ± 0.23	6.97 ± 0.25	6.99 ± 0.23	7.03 ± 0.24	7.06 ± 0.26	7.09 ± 0.27	7.12 ± 0.25	7.14 ± 0.27	7.16 ± 0.32
*P*. *ostreatus/C*. *vulgaris*	6.86 ± 0.18	6.88 ± 0.21	6.95 ± 0.24	6.98 ± 0.27	7.04 ± 0.25	7.08 ± 0.28	7.12 ± 0.21	7.05 ± 0.26	7.14 ± 0.23	7.17 ± 0.29	7.11 ± 0.24
*G*. *lucidum/S*. *obliquus*	6.81 ± 0.17	6.84 ± 0.22	6.87 ± 0.21	6.93 ± 0.26	6.99 ± 0.28	7.05 ± 0.22	7.08 ± 0.32	7.12 ± 0.24	7.16 ± 0.27	7.13 ± 0.25	7.15 ± 0.32
*G*. *lucidum/S*. *capricornutum*	6.84 ± 0.15	6.88 ± 0.26	6.92 ± 0.22	6.97 ± 0.23	7.03 ± 0.25	7.07 ± 0.25	7.09 ± 0.29	7.13 ± 0.23	7.15 ± 0.26	7.17 ± 0.29	7.13 ± 0.28

The COD of the biogas slurry can be efficiently removed during the biogas purification, which was in line with that of biogas CO_2_ removal and microalgae growth. These results were affirmed in the work of Tongprawhan *et al*.^47^, who suggested that CO_2_ fixation with microalgae was environmentally sustainable in wastewater purification. The COD reduction is attributed to the assimilation process of microalgae, which involves cell growth of microalgae and microalgal-fungal pellets, the CO_2_ uptaking of the microalgal and microalgal-fungal pellets was positively related to the microalgal cell growth and COD removal^48^. The microalgae cells assimilation requires abundant carbon from biogas slurry and biogas for producing nucleic acid^49^. Furthermore, Chisti^45^ reported that approximately half of the microalgae cell was carbon derived from CO_2_ uptaking. Especially, the synthetic materials in the pollutant removal process could be used during the microalgal autotrophic metabolism. They are acted as an enzyme activator or energy (ATP), and as the components of microalgae^50^.Therefore, the COD in biogas slurry can promote the CO_2_ removal efficiency of biogas using microalgae.

### Energy consumption economic efficiency

Table 2 also showed the energy consumption economic efficiency for biogas CO_2_ removal efficiency and the biogas slurry nutrient removal efficiency using different microalgae with different CO_2_ content. The results show that, for 35% CO_2_, *G*. *lucidum/C*. *vulgaris* has the highest energy efficiency in these five cultures. Although the TN removal of *G*. *lucidum/S*. *obliquus* was 2.3% higher than *G*. *lucidum/C*. *vulgaris*, the difference in TN removal rate was not significant (*p* > 0.05). Similarly, *G*. *lucidum/C*. *vulgaris* can achieve relatively high energy efficiency with 45% and 55% CO_2_. For the same reason, there was no significant difference between *G*. *lucidum/C*. *vulgaris* and *G*. *lucidum/S*. *obliquus* for energy efficiency of TN and TP removal with 45% CO_2_ (*p* > 0.05), as well as energy efficiency of TN removal between *G*. *lucidum/S*. *obliquus* and *G*. *lucidum/C*. *vulgaris* with 55% CO_2_ (*p* > 0.05). These results are consistent with the analysis of the microalgal growth and nutrient removal mentioned above. As a result, *G*. *lucidum/C*. *vulgaris* can achieve high energy efficiency with 55% CO_2_. The reason can be conclude that, CO_2_ can provide an important and sufficient carbon source for photosynthesis of microalgae and promote its growth with 55% CO_2_. The *Ganoderma lucidum* can provide a carrier for microalgal growth that promotes their growths. The symbiont resulted in removing nutrient in the sewage and CO_2_ in biogas efficiently^2,28,51^. According to the Eq.(3), the energy efficiency depend on the removal rate of nutrients or CO_2_. According to the results of Table 2, the removal rate of *G*. *lucidum/C*. *vulgaris* is superior to other cultures and lead to high energy efficiency.

## Conclusions

Five different microalgae-fungi had significant effects on nutrient and CO_2_ removal. The removal of pollutants and biogas purification increased as the increasing of CO_2_ content in biogas. *G*.*lucidum/C*. *vulgaris* was selected as the better biological treatment with the initial 55% CO_2_ content because of its high pollutant purification efficiency. The mean COD, TN, TP and CO_2_ removal efficiency were 68.29%, 61.75%, 64.21% and 64.68%, respectively. The analysis of the energy consumption economic efficiency demonstrated that cocultivation of microalgae and fungi experienced the highest economic efficiency.

## Methods

### Cultivation of microalgal, fungal and fungal-microalgal strains and culture conditions

Three microalgae named *C*. *vulgaris*, *S*. *obliquus and S*. *capricornutum* were used for nutrient removal and biogas upgrading because of their high biogas tolerance and fast growth rate in high nutrient concentration wastewater^2,18^. They were cultured on BG-11 medium, which was autoclaved before and contained NaNO_3_ (1500 mg L^−1^), K_2_HPO_4_·3H_2_O (40 mg L^−1^), MgSO_4_·7H_2_O (75 mg L^−1^), CaCl_2_·2H_2_O (36 mg L^−1^), ferric ammonium citrate citric acid·1H_2_O (6 mg L^−1^), EDTA-Na_2_ (1 mg L^−1^), Na_2_CO_3_ (20 mg L^−1^), and A_5_ (1 ml L^−1^). The trace elements (A_5_) consisted of H_3_BO_3_ (2860 mg L^−1^), MnCl_2_·H_2_O (1860 mg L^−1^), ZnSO_4_·7H_2_O (222 mg L^−1^), CuSO_4_·5H_2_O (79 mg L^−1^), NaMoO_4_·2H_2_O (390 mg L^−1^), and CoCl_2_·6H_2_O (49 mg L^−1^). In order to be in the exponential phase to increase their mass before the experiments, these microalgal strains were cultivated in 500 mL Erlenmeyer flasks for 7 days. All the cultivation were conducted in a controlled conditions under LED light with about 200 μmol m^−2^ s^−1^ photosynthetic photon flux density, a light-dark cycle (12 h:12 h) at 25 ± 0.5 °C in illuminating incubators (GZP-350S) obtained from Shanghai Jing Hong Laboratory Instrument Co., Ltd. (Shanghai, China). LED lamps were evenly distributed on three sides (left, right and front) of the incubators. The dry weight (DW) of these selected microalgal strains in the stock culture was nearly 66.38 ± 3.17 mg L^−1^.

Similarly, three fungal strains obtained from China General Microbiological Culture Collection Center were selected in this study for the further research as they have high growth rate and high pelletization ability (namely, *P*. *geesteranus*, *G*. *lucidum* and *P*. *ostreatus*). To form pellets, spore solutions were cultivated at 25 ± 0.1 °C for 7 d on 500 mL synthetic growth medium (glucose, 10 g L^−1^; NH_4_NO_3_, 2.0 g L^−1^; K_2_HPO_4_, 1.0 g L^−1^; NaH_2_PO_4_·H_2_O, 0.4 g L^−1^; MgSO_4_·7H_2_O, 0.5 g L^−1^; and yeast extract, 2.0 g L^−1^; pH 6.5). The obtained biomass was washed and homogenized with 100 mL of sterile distilled water in a laboratory blender. Subsequently, these obtained strains were used for the co-cultivation with microalgae.

As far as fungal-microalgal co-cultivation was concerned, microalgal suspensions (100 mL) of *C*. *vulgaris*, *S*. *obliquus*, and *S*. *capricornutum* were obtained after preparation and then each suspension was mixed with 5 mL of *P*. *geesteranus*, *G*. *lucidum* or *P*. *ostreatus* pellet suspension. The co-culture conditions for fungal–microalgal mixtures were as follows: constant light 200 μmol m^−2^ s^−1^, 25 ± 0.5 °C, artificial intermittent shaking at 160 rpm approximately for 168 h. All of the biogas upgrading and wastewater purification experiments were biologically conducted in triplicated and the daily biomass concentrations were measured during operational periods in 10 days.

### Photobioreactor

**Figure 5:**
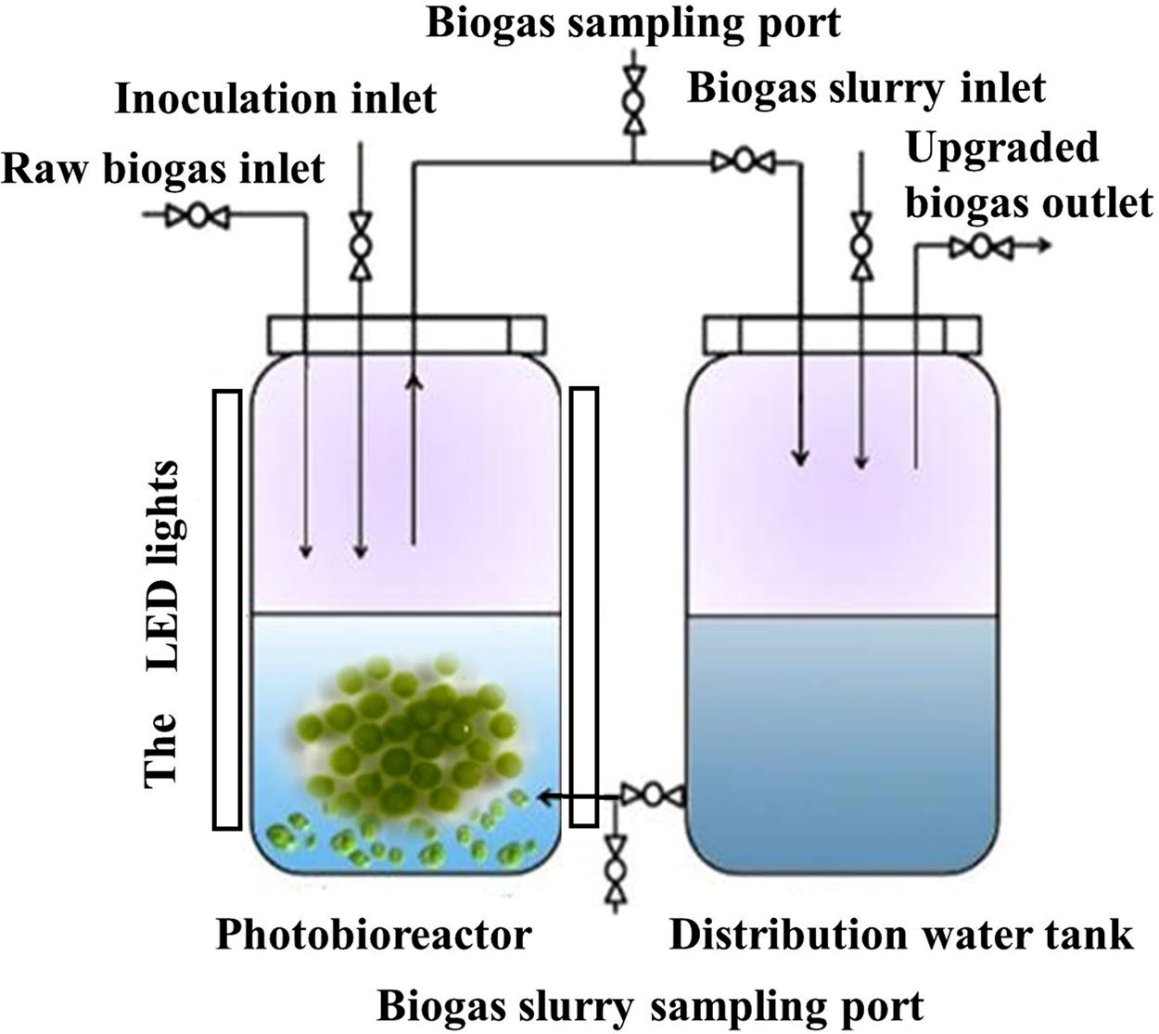
The photobioreactor experimental setup.

The photobioreactor was formed of two individual, interconnected with a glass cylinder blocks (volume = 16.8 L, height = 0.6 m; diameter = 0.2 m), filled with 14 L crude biogas and 2.8 L biogas slurry^28^ (Fig. 5). Rubber stoppers were used to seal reactors. Biogas slurry was once added to the photobioreactor from the right-cylinder block to the left, with the left-cylinder block illuminated under 200 μmol m^−2^ s^−1^. Crude biogas was fed to the system via a photobioreactor headspace.

### Biogas slurry and biogas

The CO_2_ content in synthetic biogas were 35.26 ± 2.19% (vol.%), 45.28 ± 1.92% (vol.%), 55.13 ± 3.11% (vol.%). The biogas slurry was obtained from an anaerobic digester in Hongmao Hacienda, Kunshan City, Jiangsu Province, PR China. The raw biogas slurry was pretreated by passing through a glass microfiber filter (GF/C; Whatman, USA) and ultraviolet sterilizer (SKW-UVU01; SKYUV Water Treatment Co. Ltd, China) for 2 minutes to prevent potential interference from sediment and some microorganisms^18^. The characteristics of the raw biogas slurry before and after pretreatment were listed in Table 4, which revealed that the characteristics of biogas slurry almost unchanged before and after pretreatments.

**Table 4:** The basic characteristics of biogas slurry.

Parameter	Before pretreatment	After pretreatment
pH	6.84 ± 0.16	6.91 ± 0.18
DO	5.76 ± 0.41	5.59 ± 0.27
DIC(mgL^−1^)	831.55 ± 19.38	809.21 ± 22.04
COD(mgL^−1^)	1024.36 ± 30.43	997.57 ± 27.39
TN(mgL^−1^)	209.35 ± 17.19	202.07 ± 15.66
TP(mgL^−1^)	22.48 ± 3.71	20.92 ± 2.37

### Experimental procedure

According to our previous studies, mono-microalgal strain *C*. *vulgaris* and mono-fungal stain *G*. *lucidum* already showed great ability on biogas upgrading and simultaneously biogas slurry nutrients removal by itself in the bioreactor^21,23,28^. Hence, three above-mentioned fungal strains were co-cultivated with *C*. *vulgaris*, and three selected microalgal strains were co-cultivated with *G*. *lucidum* in this study for the further study. In view of one mixture was double counted, there are five fungal-microalgal mixtures were co-cultivated for next step in this experiment, such as *P*. *geesteranus/C*. *vulgaris*, *G*. *lucidum/C*. *vulgaris*, *P*. *ostreatus/C*. *vulgaris*, *G*. *lucidum/S*. *obliquus and G*. *lucidum/S*. *capricornutum*.

Detailed procedures were as follows based on research design above: 100 mL of microalgal suspensions of *C*. *vulgaris*, *S*. *obliquus and S*. *capricornutum* (about 118 mg L^−1^ of all the dry weight) were cultured, then each suspension was mixed with 5 mL of *P*. *geesteranus*, *G*. *lucidum* and *P*. *ostreatus* pellet suspension (about 83 mg L^−1^ of dry weight). The initial density of the microalgae co-cultivated with fungal cells was maintained at about 123.52 ± 3.46 mg·L^−1^ for the five fungal–microalgal pellets. The following conditions were used: the light intensity was 200 μmol m^−2^ s^−1^, the experimental period was 10 d, the temperature was 25 ± 0.5 °C and the 1ight:dark cycles was 12 h:12 h. The growth rates, mean daily productivity, nutrient removal and CO_2_ content with different fungal-microalgal co-cultivation types were evaluated daily and the optimal CO_2_ concentration was selected by analyzing the economic efficiencies of the biogas CO_2_ and the biogas slurry nutrient removal.

### Sampling and analyses

The biogas slurry in photobioreactors was sampled daily for determination of COD, total nitrogen (TN) and total phosphate (TP). The biogas was sampled for component analysis (CH_4_, CO_2_, O_2_ and H_2_O, v/v) using a circulating gas analyzer (GA94; ONUEE Co. Ltd, China). Dry weights of microalgae were measured through exsiccation after being filtered with a glass microfiber filter (GF/C, Whatman, USA). The filtrates were used for nutrient determination according to the standard methods^52^.

Biogas CO_2_ and total biogas slurry nutrient removal efficiency (RE, %) was calculated based on the following equation:

1 $$RE=(1-\frac{{C}_{i}}{{C}_{0}})\times 100$$

where *C*_i_ is the biogas CO_2_ content or total nutrient concentration (g L^−1^) in cultures at time *t*_i_ and *C*_0_ is the initial biogas CO_2_ content or total nutrient concentration (g L^−1^) at time *t*_0_ (day).

Specific growth rates (*μ*) were derived from the growth phase using the following equations:

2 $$\mu =\frac{(\mathrm{ln}\,{{\rm{D}}}_{t}-\,\mathrm{ln}\,{{\rm{D}}}_{0})}{t}\to \,\mathrm{ln}\,{{\rm{D}}}_{t}=\mu t+\,\mathrm{ln}\,{{\rm{D}}}_{0}$$

where *D*_i_ stand for the biomass concentration (g L^−1^) at time *t*_i_ (d) and *D*_0_ is the biomass concentration (g L^−1^) at time *t*_0_ (d).

The CO_2_ or biogas slurry nutrient removal economic efficiency was evaluated based on the following equation:

3 $$E=\frac{R}{kTP}$$

where *E* is the biogas CO_2_ or biogas slurry nutrient removal economic efficiency (USD^−1^), *R* is the removal efficiency of pollutant (%) in Eq. (1), k is the electric power charge per unit of power consumption (USD kW^−1^ h^−1^), which is around 0.645 RMB kW^−1^ h^−1^ in local, i.e. around 0.097 USD kW^−1^ h^−1^; *T* is the light application time (h), and *P* is the LED electric quantity (W).

### Statistical analyses

Statistic analysis was carried out using Statistic Package for Social Science (SPSS, V19.0). One-way analysis of variance (ANOVA) was used to determine whether the impact of various factors on the test indicators is significant. Duncan’s multiple range tests was used to analyze the significant difference between groups. The value *p* = 0.05 was regarded as the threshold for statistical significance.
